# Repurposing Approved Drugs for Guiding COVID-19 Prophylaxis: A Systematic Review

**DOI:** 10.3389/fphar.2020.590598

**Published:** 2020-12-14

**Authors:** Bruno Silva Andrade, Fernanda de Souza Rangel, Naiane Oliveira Santos, Andria dos Santos Freitas, Wagner Rodrigues de Assis Soares, Sérgio Siqueira, Debmalya Barh, Aristóteles Góes-Neto, Alexander Birbrair, Vasco Ariston de Carvalho Azevedo

**Affiliations:** ^1^ Laboratório de Bioinformática e Química Computacional, Departamento de Ciências Biológicas, Universidade Estadual do Sudoeste da Bahia (UESB), Jequié, Brazil; ^2^ Programa de Pós-graduação em Genética e Biologia Molecular, Universidade Estadual de Santa Cruz, Ilhéus, Brazil; ^3^ Departamento de Saúde II, Universidade Estadual do Sudoeste da Bahia, Jequié, Brazil; ^4^ Centre for Genomics and Applied Gene Technology, Institute of Integrative Omics and Applied Biotechnology (IIOAB), Purba Medinipur, India; ^5^ Laboratório de Biologia Molecular e Computacional de Fungos, Departamento de Microbiologia, Instituto de Ciências Biológicas, Universidade Federal de Minas Gerais (UFMG), Belo Horizonte, Brazil; ^6^ Departamento de Patologia, Instituto de Ciências Biológicas, Universidade Federal de Minas Gerais (UFMG), Belo Horizonte, Brazil; ^7^ Laboratório de Genética Celular e Molecular, Departamento de Biologia Geral, Instituto de Ciências Biológicas, Universidade Federal de Minas Gerais, Belo Horizonte, Brazil

**Keywords:** SARS-CoV-2, prophylaxis, antiviral, drug repurposing, COVID-19

## Abstract

The SARS-CoV-2 outbreak originally appeared in China in December 2019 and became a global pandemic in March 2020. This infectious disease has directly affected public health and the world economy. Several palliative therapeutic treatments and prophylaxis strategies have been used to control the progress of this viral infection, including pre-(PrEP) and post-exposure prophylaxis. On the other hand, research groups around the world are still studying novel drug prophylaxis and treatment using repurposing approaches, as well as vaccination options, which are in different pre-clinical and clinical testing phases. This systematic review evaluated 1,228 articles from the PubMed and Scopus indexing databases, following the Kitchenham bibliographic searching protocol, with the aim to list drug candidates, potentially approved to be used as new options for SARS-CoV-2 prophylaxis clinical trials and medical protocols. In searching protocol, we used the following keywords: “Covid-19 or SARS-CoV-2” or “Coronavirus or 2019 nCoV,” “prophylaxis,” “prophylactic,” “pre-exposure,” “COVID-19 or SARS-CoV-2 Chemoprophylaxis,” “repurposed,” “strategies,” “clinical,” “trials,” “anti-SARS-CoV-2,” “anti-covid-19,” “Antiviral,” “Therapy prevention *in vitro*,” in cells “and” human testing. After all protocol steps, we selected 60 articles that included: 15 studies with clinical data, 22 studies that used *in vitro* experiments, seven studies using animal models, and 18 studies performed with in silico experiments. Additionally, we included more 22 compounds between FDA approved drugs and drug-like like molecules, which were tested in large-scale screenings, as well as those repurposed approved drugs with new mechanism of actions. The drugs selected in this review can assist clinical studies and medical guidelines on the rational repurposing of known antiviral drugs for COVID-19 prophylaxis.

## Introduction

The SARS-CoV-2 outbreak originally appeared in China in December 2019 and became a global pandemic in March 2020 (Xie et al., 2020). This infectious disease made a direct impact on global public health, and is still impairing the world economy (World Health Organization, 2020). In order to minimize and prevent the advance of COVID-19 and its effects, the world scientific community has been doing an unprecedented race in many research fields, resulting in many discoveries in viral biology, disease physiopathology, and new more effective and cost-beneficial therapeutic options to be used for the treatment of people affected by the new virus (Vellingiri et al., 2020).

Prophylactic drugs can be used both to block the pathogen’s infectious cycle and/or to boost host immunity (Glushkov et al., 1999). There are two main categories of prophylaxis (I) pre-exposure prophylaxis (PrEP), which considers that treated individuals that had no contact with the pathogen (II) post-exposure prophylaxis (PEP), which includes individuals that may have been infected (e.g. contact with patients) but have not exhibited the disease symptoms (Zhang et al., 2020). These two models of prophylactic studies have been extensively used in endemic viral pathologies with high transmissivity, such as HIV (Krakower et al., 2015). Additionally, both methods exhibited success for other viral diseases with great global health impact (Mayer et al., 2015). PrEP and PEP have proven to be extremely effective strategies in viral transmission control for patients inside certain risk groups, such as those with comorbidities, and health professionals directly exposed to the risk of acquiring and transmitting Covid-19 (Rockstroh et al., 2020).

Prophylactic antiviral treatment is an important approach for rational drug administration since it can be used to block the disease evolution and to spread and reduce the risks of side and adverse effects, as well as toxicity in patients (Beigel et al., 2019; Cheng, 2019). Because of SARS-CoV-2 high degree of transmissivity, novel therapeutic ways that can reach the affected patients faster became necessary (Kang et al., 2020). Among many therapeutic strategies, drug repurposing has been reaching significant results against some pathogens (Cheng et al., 2016; Cheng, 2019). Drugs approved for human diseases can be repurposed for new targets in order to speed up the process of implementing these compounds in clinical protocols for the treatment and prophylaxis in the acute phase of viral diseases. Moreover, this approach is instrumental in preventing the viral transmission to healthy individuals (Zhou et al., 2020).

Currently, several preclinical studies, such as *in silico*, *in vitro* and *in vivo* trials have been guiding clinical decisions in choosing the best drug options for the treatment and prophylaxis against SARS-CoV-2 (Fragkou et al., 2020). Therefore, different drug classes with prophylactic properties have been repositioned in order to guarantee protection against viral transmission (Zhou et al., 2020). This could lead to an interesting strategy targeting COVID-19 since it can be used as an additional barrier to viral spreading, as well as preventing disease evolution, especially for patients inside the risk groups. In this review, we present a systematic analysis of the main antiviral drug agents for many diseases, which can be proposed as new prophylaxis in clinical trials against SARS-CoV-2 infection and other therapeutic interventions.

## Material and Methods

This systematic review was conducted in five stages: planning, bibliographic search, initial selection, final selection, summary of data and results. All of these steps were performed based on the bibliographic search protocol model developed by Kitchenham (2004). We used two indexing databases for the bibliographic search: PubMed (www.ncbi.nlm.nih.gov) and Scopus (www.scopus.com), in order to retrieve papers related to the proposed theme of this review, and considered publications until June 30, 2020. On the PubMed database searching, we considered 38 strings with the terms: “Covid-19,” “SARS-CoV-2,” “Coronavirus,” “2019 nCoV” and “Prophylaxis,” combined with 38 drug names. The Scopus database search produced eight strings, constructed using the following words: prophylaxis, prophylactic, pre-exposure, COVID-19, SARS-CoV-2, Chemoprophylaxis, repurposed, strategies, clinical, trials, anti-SARS-CoV-2, anti-covid-19, antiviral, therapy prevention *in vitro*, in cells, and human testing. The detailed steps on sorting publications are described in the Supplemental Material S1.

The publications retrieved were imported to MEDLINE (PubMed) and BIBTEX (Scopus) formats, and submitted to the StArt (State of the Art through Systematic Review) program v. 3.3 Beta 03 (Fabbri et al., 2016), developed by the Federal University of São Carlos (UFSCar), and available for download on http://lapes.dc.ufscar.br/tools/start_tool. Furthermore, we excluded duplicated records, and then all the preselected publications were entered into an Excel spreadsheet model for the next steps.

The initial selection of publications was based on the inclusion and exclusion criteria described in the following protocol. Initially, all the review articles, case studies, clinical guidelines, research strategies, short communications and unfinished studies were excluded. In a second phase, we included only publications of *in vitro*, *in vivo* research, and randomized clinical studies on the use of drugs for pre- and post-exposure prophylaxis for the treatment of COVID-19. Additionally, until the end of the review process, we included novel pre-clinical and clincal SARS-CoV-2 drug repurposing studies as a way of complementing the discussion about drug prophylaxis against COVID-19.

The corresponding metadata of used drugs for each study, test phases (I, II, and III), number of patients, cell lineages used in *vitro* tests, as well as *in vivo* test details were extracted from each accepted publication, and this information is presented in the Supplemental Material S2, as well as all 1,288 reference articles used in this work are reported in the Supplemental Material S3.

Additional information about drug efficacy, half-life, toxicity, interactions, and side effects were obtained from the public domain database Drugbank (https://www.drugbank.ca/).

## Results

**FIGURE 1:**
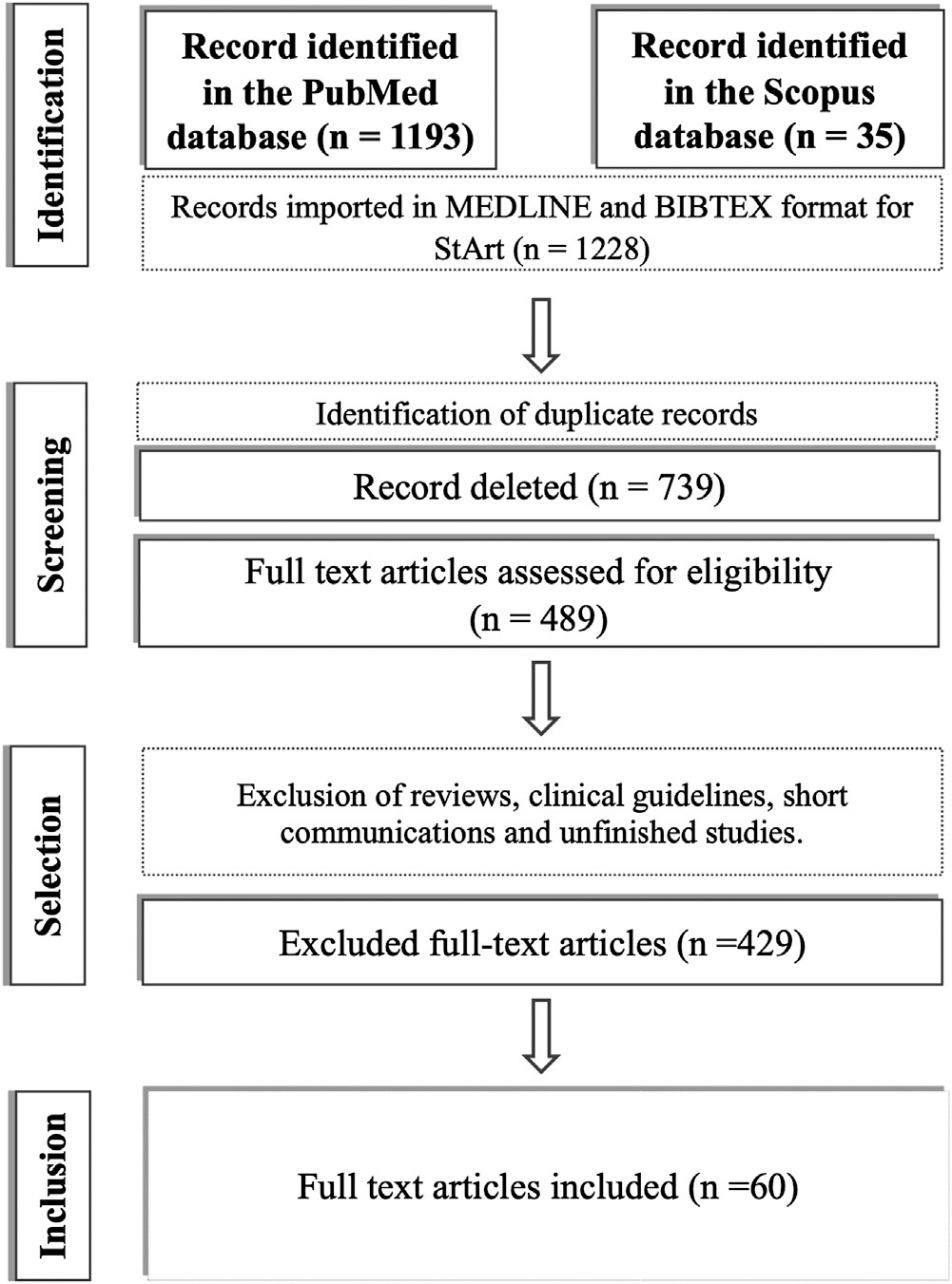
Detailed systematic review protocol and results for both PubMed and Scopus indexing bases with all the steps for inclusion and exclusion of potential prophylaxis articles.

In this review, we reported a significant number of articles with human clinical studies, *in vivo* animal experiments, *in vitro* cell studies, and *in silico* approaches, mainly for drug repurposing strategies. Furthermore, we evaluated literature material of 1,228 article records. The screening, selection, and exclusion processes of all the publications are detailed in Figure 1.

After all the filtering steps included in our systematic protocol, we selected 60 articles to describe possible prophylaxis options for preclinical and clinical studies against SARS-CoV-2. The analysis of articles’ contents indicated that: 15 studies were done with clinical data; 22 studies used *in vitro* approaches against pathogenic virus strains responsible for airway and pulmonary infections, such as influenza and SARS-CoV-2; seven studies used animal models; and 18 studies performed *in silico* experiments against viral targets. Furthermore, we included 37 complementary articles discussing 23 drug mechanism of action as additional prophylaxis options.

Usually, the studies did not discuss drug half-life, as well as other pharmacokinetic parameters, such as Cmax, Tmax, and renal clearance. All the drug doses reported were those used for daily treatment for parasitic and viral infections. On the other hand, by using the information from the *in vitro* tests, it is possible to predict a range of inhibitory concentrations, which could assist in extrapolating the concentration parameters in clinical studies with humans. Nonetheless, extrapolation of plasma dose and concentration should be assessed and monitored in blood plasma, as prophylactic studies have shown that some broad-spectrum antiviral drugs should be administered in concentrations greater than those provided for clinical protocols. Thus, it is possible to create more effective therapeutic responses against COVID-19; however, increasing doses and adjustment may induce potential adverse and side effects, as well as toxic and drug interactions, which has not been reported in most of the clinical studies considered in this review. All the reported drugs for the accepted papers are included in Table 1.

**TABLE 1:** Screened drugs with potential for prophylaxis studies, and their correspondent number of citations and mechanism of action.

	Drug	Citations	Mechanism of action
1	Aciclovir	World Health Organization (2020)	Nucleoside analog
2	Amantadine	World Health Organization (2020)	Interferes with transmembrane M2 protein
3	Amprenavir	World Health Organization (2020)	Protease inhibitor (HIV)
4	Baloxavir marboxil	Xie et al. (2020)	Endonuclease inhibitor—inhibits the initiation of mRNA synthesis
5	Darunavir	Zhang et al. (2020)	Second generation protease inhibitor
6	Entecavir	World Health Organization (2020)	Guanine analogue (HCV)
7	Faldaprevir	World Health Organization (2020)	HCV protease inhibitor
8	Faviparivir	Beigel et al. (2019)	Prodrug of a purine nucleotide, favipiravir ribofuranosyl-5′-triphosphate—RNA polymerase inhibitor
9	Galidesivir	Mayer et al. (2015)	Protease inhibitor—Adenine analog
10	GS-441524	Xie et al. (2020)	Adenisin nucleoside analog
11	Indinavir	World Health Organization (2020)	HIV protease specific inhibitor
12	Lopinavir	Beigel et al. (2019)	Aspartic acid protease (HIV) inhibitor
13	Nelfinavir	Vellingiri et al. (2020)	Protease inhibitor
14	Oseltamivir	Vellingiri et al. (2020)	Active neuraminidase inhibitor
15	Pleconaril	Xie et al. (2020)	Viral capsid inhibitor
16	Remdesivir	Cheng (2019)	Prodrug—active nucleoside analog C-adenosine triphosphate—(Ebola)
17	Ribavirin	Kang et al. (2020)	Nucleoside analogue (guanine)—inhibits viral RNA-dependent RNA polymerase
18	Sofosbuvir	Glushkov et al. (1999)	Nucleoside analog—hepatitis C virus NS5B polymerase inhibitor
19	Tenofovir	Glushkov et al. (1999)	Acyclic nucleoside analog adenosine monophosphate
20	Tipranavir	Xie et al. (2020)	HIV protease enzyme inhibitor
21	Umifenovir	Zhou et al. (2020)	Hemagglutinin inhibitor (influenza)
22	Zanamivir	World Health Organization (2020)	Neuraminidase inhibitor

**FIGURE 2:**
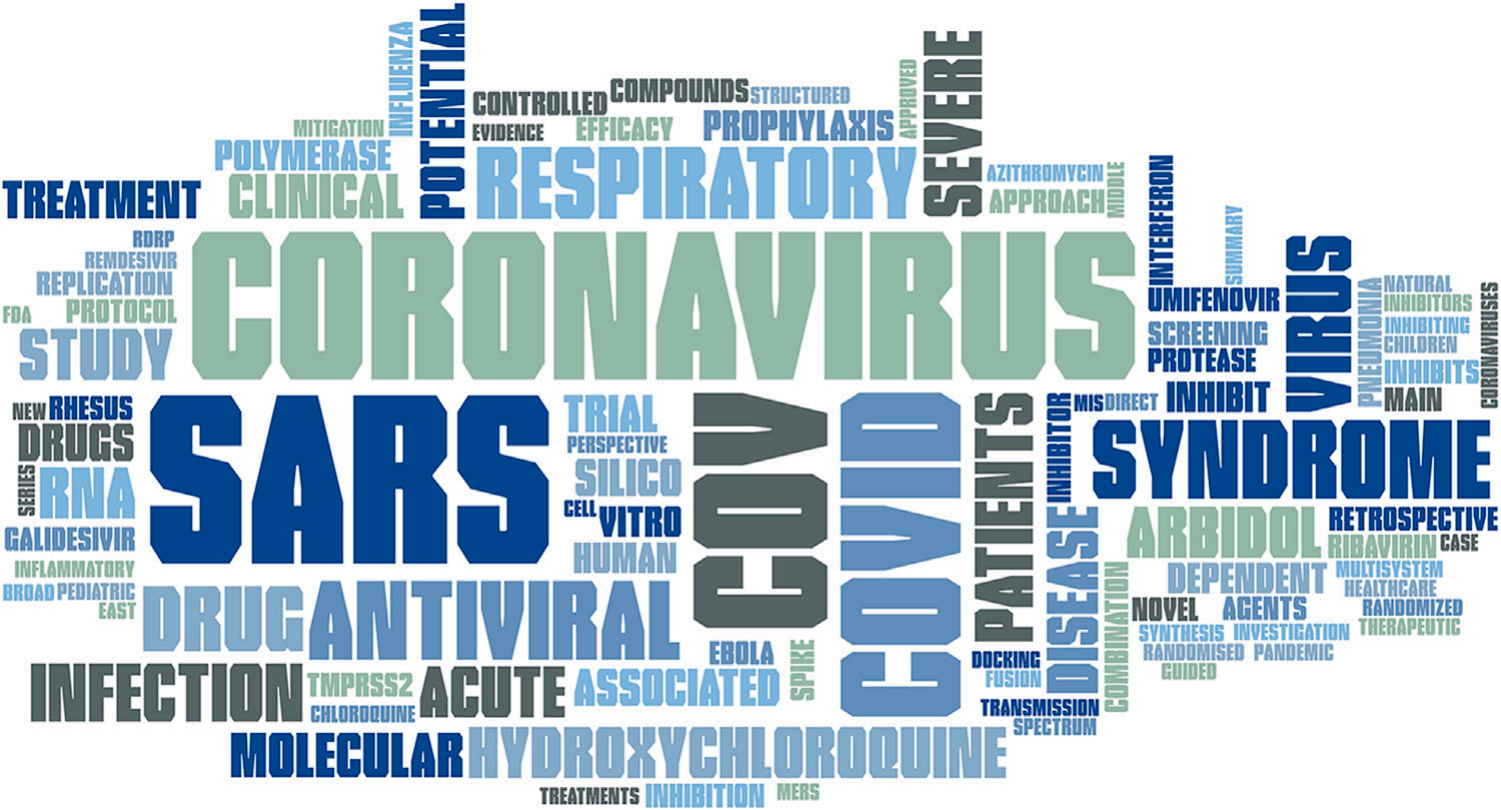
Word map reflecting the most cited terms for all the evaluated articles used in the review processes.

**FIGURE 3:**
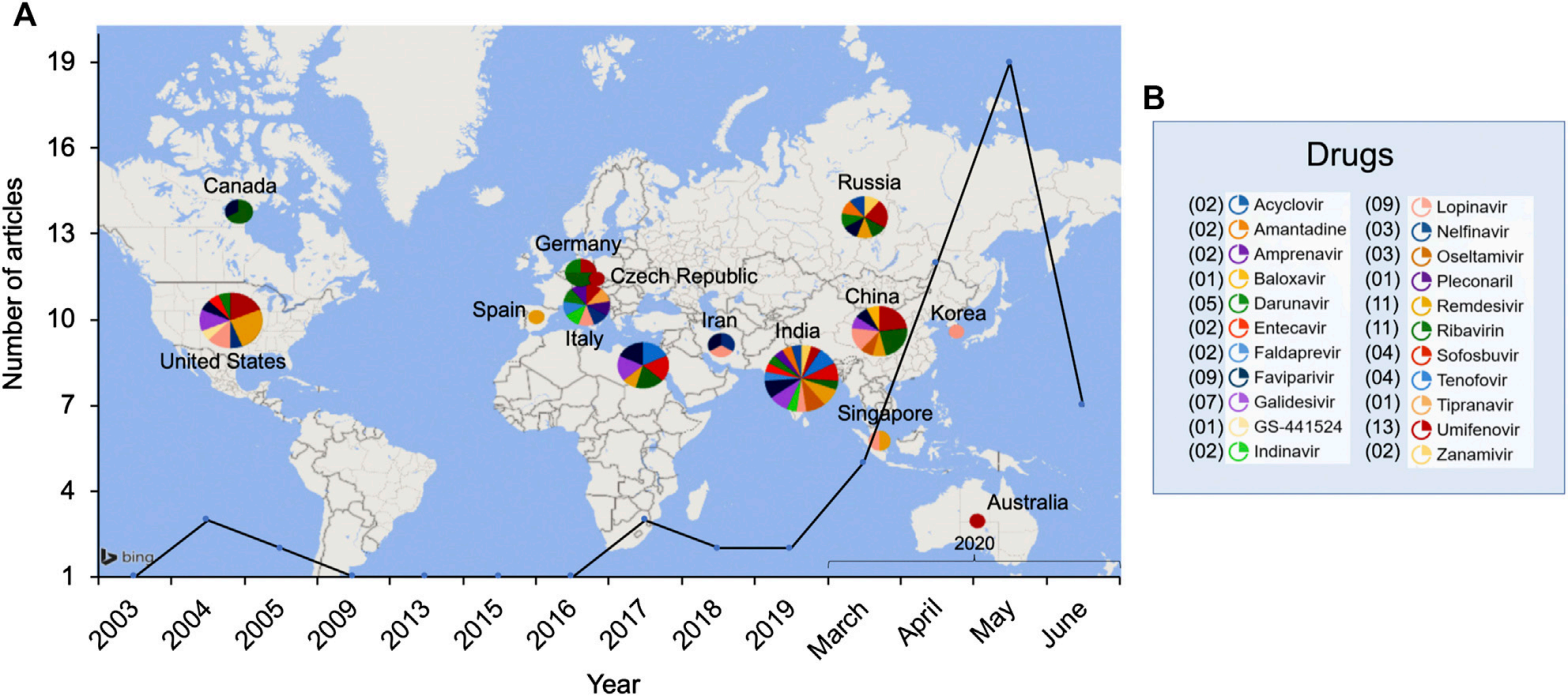
Antiviral research distribution for the 22 selected drugs. **(A)** Most relevant countries for the selected drugs between 2003 and 2020. **(B)** Number of times cited per drug.

After completing the table, we generated a word map that reflects most important drugs and terms according to the frequency they appear in all the evaluated articles (Figure 2), as well as we showed a worldwide research distribution map (Figure 3) with all the antiviral drugs cited in the Table 1. Despite other terms, such as hydroxychloroquine and some immune, antibody, and anti-parasitic drugs also appeared in this word map, we only considered drugs with antiviral action. Furthermore, these other terms can be explored in other review and research studies.

## Discussion

In this section, we make a brief description of each selected drug from Table 1, and their mechanisms of action in order to support a possible repurposing of these approved drugs as new candidates on clinical trials studies as antiviral options against SARS-CoV-2, mainly for prophylaxis but not only restricted to it.

*Amantadine* is an M2 (Matrix protein 2) viral membrane protein inhibitor, necessary for the efficient release of the viral genome during virus entry (Jing et al., 2008). This drug has been used in the prophylactic or symptomatic treatment of influenza A but also acts as an antiparkinsonian (Lamb et al., 2005). The main hypothesis indicates that amantadine could interfere with the gene expression of endosomal cysteine protease (cathepsin L or B) in SARS-CoV-2 (Smieszek et al., 2020).

*Amodiaquine* is an aminoquinoline antimalarial drug, which has been used in other antiviral studies as a protease inhibitor, such as DENV2 and West Nile virus NS2B-NS3 protease using BHK-21 and Vero cells (Boonyasuppayakorn et al., 2014; D’alessandro et al., 2020), and Ebola virus (EBOV) by blocking viral replication in Huh seven and Vero E6 cells with IC50 = 2.8–3.2 μM and 9.5–11 μM, respectively. Additionally, amodiaquine present a synergic effect against viral replication of the SARS-CoV-2 in Vero E6 cells when it is combined with nelfinavir, and it presents higher synergic index when copared with other antimalarial drugs such as chloroquine, hydroxychloroquine, quinacrine and mefloquine (Ianevski et al., 2020). One theoretical pharmachophore modeling study published in Chemrxiv reinforces a possible action of this aminoquinoline inhibitor against the SARS-CoV-2 virus, with a mechanism of blocking its main protease with a theoretical Ki of 0.073 μM(Acharya, 2020).

*Amprenavir* is a known inhibitor of the HIV protease enzyme, acting on the prevention of the gag-pol polyprotein cleavage and resulting in the formation of immature and non-infectious viral particles (Fung et al., 2000). Generally, HIV protease inhibitors are used in combination with at least two other anti-HIV drugs (Sadler et al., 2020). Recent *in vitro* studies demonstrated that amprenavir exhibited a considerable degree of inhibition against SARS-CoV-2 (Mugisha et al., 2020).

*Apilimod* is a known interleukin-13/23 production inhibitor by acting on the phosphatidylinositol-3-phosphate 5-kinase (PIKfyve) enzyme (Cai et al., 2013), as well is a safety drug for humans with a profile at doses of up to 125 mg twice daily and a peak serum concentration of 0.265 ± 0.183 μM (Huang et al., 2020). This drug was tested in *vitro* studies against EBOV and Marburg virus (MARV) using Vero E6, Huh seven cells and macrophages (hMDMs), and it was found that this inhibitor was capable of blocks both viral infection in all cell types (Nelson et al., 2017), as well as blocked EBOV particle entry (Nelson et al., 2017; White et al., 2018) and trafficking in cell cytoplasm (Nelson et al., 2017), with low IC_50_ values (Nelson et al., 2017; White et al., 2018). Additionally, apilimod has blocked Zaire ebolavirus (ZEBOV) and SARS-CoV-2 *in vitro* replication by acting on the PIKfyve kinase and reducing the intracellular trafficking of viral particles, as well as viral entry using Vero E6 cells with IC_50_ of 10  nM (Kang et al., 2020). Furthermore, a large-scale drug repurposing study apilimod was responsible for blocking viral replication in human pneumocyte-like cells derived from induced pluripotent stem cells with IC_50_ raging from 0.088 to 0.012 μM, as well as exhibited antiviral activity in a primary human lung explant model (Huang et al., 2020). Other study with Vero E6 cell viral replication monitored by QRT-PCR assays, indicated that apilimod potentially decreased the amount of SARS-CoV-2 RNA in cell culture supernatants, with an IC_50_ of 12  mM and low cell toxicity (Shema Mugisha et al., 2020).

*Baloxavir marboxyl* is an influenza A and B antiviral, which inhibits the cap-dependent endonuclease necessary for viral replication, and this is the first representative of influenza-type PB2 inhibitors. Baloxavir is under investigation in the clinical trial NCT04327791 since March 2020, and it has been used in a combined therapy with oseltamivir 1 with hospitalized patients with influenza infections (Reina and Reina, 2019).

*Darunavir* is an HIV protease inhibitor used against HIV infection, especially indicated to patients with a previous antiretroviral therapy history (Lu et al., 2020). *In silico* studies indicated that this drug could function as a protease inhibitor, as well as also interact with the 3C-like proteinase. Additionally, darunavir can also bind to the proteins of the SARS-CoV-2 replication complex (Beck et al., 2020). *In vitro* data for this drug showed potential for SARS-CoV-2 inhibition (Yamamoto et al., 2020). On the other hand, darunavir has not showed antiviral activity against SARS-CoV-2 at clinically relevant concentrations yet (De Meyer et al., 2020), but four other clinical trials are between phases 3 and 4 in order to evaluate the efficacy, safety, and pharmacokinetic characteristics of this drug in combination with other antiviral and anti-parasitic compounds against COVID-19 (Ciesek, 2020; Elmekaty, 2020; Lu, 2020; Gilead Sciences, 2020).

*Entecavir* is a guanine analogue that directly inhibits the replication process of hepatitis B virus (HBV) by blocking its reverse transcriptase mechanism (Huynh et al., 2020; Shah et al., 2020). This drug was considered a possible inhibitor of both RNA-dependent RNA polymerase and the main protease enzyme, from SARS-CoV and SARS-CoV-2 viruses (Shah et al., 2020).

*Faldaprevir* is a known Hepatitis C virus (HCV) NS3-4A protease inhibitor (HCV) (Kanda et al., 2015; Kanda et al., 2015). Its effectiveness was also confirmed when combined with other drugs such as pegylated interferon alfa-2a and ribavirin for chronic HCV infection treatment (Sulkowski et al., 2013). *In silico* experiments indicated a possible mechanism of action of faldaprevir for inhibiting the new coronavirus enzymes (Böcher et al., 2020; Shah et al., 2020), suggesting that this drug could be tested in the next preclinical and clinical trials.

*Faviparivir* is a pyrazine analogue that acts as a prodrug and inhibits the RNA-dependent RNA polymerase (RdRp) and, consequently, blocks viral transcription and replication (de Farias et al., 2017; Furuta et al., 2017). This drug was approved for therapeutic use in Japan in 2014 for influenza viruses. Nevertheless, because RdRp catalytic domain is conserved among several types of RNA viruses, its mechanism of action supports a wide spectrum of viral targets such those against Ebola and, more recently, SARS-CoV-2. (Furuta et al., 2017; Madelain et al., 2017; Raoul, 2020).

*Galidesivir* is an adenosine analog acting through the mechanism of inhibiting the viral RNA polymerase (Zhang et al., 2020). In this case, the nucleoside analog (NA) is incorporated into the viral RNA to exhibit its antiviral activity. Moreover, another mechanism of action is the recognition of this nucleotide analog as a substrate by the viral RNA polymerase, which blocks the RNA replication. This drug was initially used for treating HCV infections, but it has been reported as also efficient against Ebola, Zika and Yellow Fever viruses (De Clercq, 2019; Elfiky, 2020). Other NAs, such as remdesivir, favipiravir, and ribavirin were reported to have efficacy against SARS-CoV-2 by blocking RdRp activity (Zhang et al., 2020).

*GC376* is a dipeptidyl bisulfite adduct salt that has been early used in cell-based inhibition assays against the picornavirus-like supercluster (picornaviruses, caliciviruses, and coronaviruses), with its mechanism of action on the inhibition of viral 3C-like proteases (Kim et al., 2012). This drug was used in a clinical study with a fatal coronavirus infection, caused by the feline infectious peritonitis virus (FIPV), and the authors have reported that GC376 significantly reduced viral load and symptoms, decreased the viral RNA levels in the macrophages from the cats that received the antiviral treatment, as well as returned all the individuals to their normal conditions by clinical observations and laboratory testing (Chang et al., 2016). Furthermore, it was reported that GC376 was capable to block the 3C-Like protease from the porcine epidemic diarrhea virus (PEDV) in cell-based assays using Vero cells, and they included a protein-inhibitor complex crystallization. In this case, the authors verified that in cell-based assays the IC_50_ was 1.11± 1.13 μM (Pedersen et al., 2020). Recently, this inhibitor has been tested against the SARS-CoV-2 in studies using fluorescence resonance energy transfer (FRET)-based screening assays targeting the Mpro enzyme, with molecular docking confirmation, as well as checking antiviral effects in infected Vero E6 cell cultures with an EC50 of 0.91± 0.03 μM (Hung et al., 2020). Other studies have reported GC376 as potent SARS-CoV-2 3C-like protease inhibitor in comparison to other drugs, such as Boceprevir, and describing their mechanism of interaction (Fu et al., 2020). In addition, other study compared the efficacy of the GC376 by generating its analogue the GC373 using FRET and cell-based studied, and including protein crystallization. The authors reported both molecules acted as potent inhibitors against SARS-CoV-2 Mpro, and presenting EC_50_ of 1.5 μM for GC373 and for 0.92 μM GC376 (Vuong et al., 2020).

*GS-441524* is a remdesivir metabolite with activity against SARS-CoV-2 Mpro in molecular docking studies. In addition, it exhibited potent antiviral activity against several RNA viruses, including SARS infections (Cho et al., 2012). Besides, this drug could provide synergistic effects in combination with other RdRp antagonist drugs (Huynh et al., 2020).

*Lopinavir* is an antiretroviral protease inhibitor used in combination with other drugs for the HIV-7 treatment (Kim et al., 2020), such as ritonavir (Wada et al., 2020), which is a peptidomimetic inhibitor designed for inhibiting HIV-1 protease and is currently under investigation for the treatment of COVID-19 (De Clercq, 2009). Clinical studies indicated a potential benefit for patients infected with SARS-CoV-2 treated with lopinavir in the early stage of the disease (Li et al., 2020; Lim et al., 2020; Wu et al., 2020). Moreover, some authors suggested a protective effect of lopinavir on post-exposure prophylaxis for Middle East Respiratory Syndrome (MERS) (Li et al., 2020), as well as in combination with other antivirals for benefiting the treatment of SARS and MERS, including the reduction of acute respiratory distress syndrome (ARDS) incidences and mortality during early treatment (Chan et al., 2003).

*Nelfinavir* is an antiretroviral protease inhibitor with *in vitro* activity against the SARS-CoV 3CL protease, and *in silico*/*in vitro* activity against SARS-CoV replication (Yuen et al., 2005). *In silico* experiments indicated nelfinavir as a SARS-CoV-2 Mpro inhibitor, which could act in combination with cepharanthine on the control of disease progression and transmission risk (Chow et al., 2004; Yamamoto et al., 2004; Yuen et al., 2005). *In vitro* findings showed that cepharanthine reduced coronavirus cell entry (Yamamoto et al., 2019; Ohashi et al., 2020). Additionally, clinical data suggest that nelfinavir exhibits good pharmacokinetics characteristics in humans, and, thus, could be a potential drug candidate prophylaxis and treatment for COVID-19 patients (Bimonte et al., 2020; Yamamoto et al., 2020).

*Oseltamivir* is an influenza A and B approved drug with action inhibiting the viral neuraminidase, which decreases the release of viral particles from host cells and reduces viral spread in the respiratory tract (Rosa and Santos, 2020; Shah et al., 2020). This drug was used in the initial months of the COVID-19 outbreak, whether combined or not with antibiotics and corticosteroids, as well as with multiple combinations with chloroquine and favipiravir clinical trials. It is important to observe that clinical trials with oseltamivir at concentrations lower than 100 μM showed no apparent *in vitro* antiviral effect against the SARS-CoV-2 (Choy et al., 2020). In a clinical trial article, the authors indicated that this drug is not effective inhibiting SARS-CoV-2 even at its highest concentration; however, they do not show details of the trial, which is important to correctly determine the stage of infection at which the drug was administered and its effectiveness against COVID-19 (Rosa and Santos, 2020).

*PF-00835231* is a ketone-based designed for inhibiting the SARS-CoV-1 virus (Yen et al., 2004). Recently, two studies revealed this drug as a potent inhibitor which were tested by FRET assay and for antiviral activity in Vero E6 cells with an IC50 0.00027 ± 0.0001 μM for 3C-like protease (Hoffman et al., 2020), as well as demonstrated to be statistically more potent than remdesivir in assays with infected SARS-CoV-2 A549^+ACE2^ cells with an EC_50_ of 0.221 μM at 24 h, and 0.158 μM at 48 h without detectable cytotoxicity (de Vries et al., 2020).

*Remdesivir* is a nucleoside analog inhibitor of RNA polymerases with a large viral spectrum (Gordon et al., 2020), originally developed for the treatment against Ebola (Cheng et al., 2020), exhibiting antiviral effects against filoviruses, paramixoviruses, pneumoviruses, and coronaviruses (Wang et al., 2020). This drug has been tested both *in vitro* and *in vivo* experiments (mice and Rhesus monkeys) against SARS-CoV2 (Flanigan et al., 2020; Grein et al., 2020). Furthermore, clinical trials have been performed with SARS-CoV-2 infected adults and children in different dose ranges, and it has been demonstrating low toxicity (Hennon et al., 2020; Maharaj et al., 2020). Additionally, double-blind, randomized, multicenter clinical studies observed a significant improvement in the reduction of viral load during the infection but without a considerable reduction in the mortality rate compared to patients who received placebo in the same period (Wang et al., 2020). Moreover, *in vitro* cell culture Vero-E6 tests showed antiviral activity of this drug in the post-entry stage of the cells, with an EC50 of 1.76 μM in EC50 (Wang et al., 2020).

*Ribavirin* is a guanosine analogue antiviral with activity against DNA and RNA viral polymerases that has been showing promising results against SARS-CoV-2 (Alhazzani et al., 2020; Khalili et al., 2020; Yousefi et al., 2020; Zhang et al., 2020). Although this compound is established among the first five antiviral drugs tested *in vitro* against SARS-CoV-2 due to its promising results against previous SARS and MERS infections (Morgenstern et al., 2005; Wohlford-Lenane et al., 2009; Khalili et al., 2020), some authors indicated a need of dose reduction on the new coronaviruses treatment (McCray et al., 2020). The prophylactic use of this drug includes the association with lopinavir and ritonavir, or interferon-α (INF-α), instead of monotherapy. Studies about ribavirin in the SARS-CoV-2 treatment still lack information about mechanisms of action, dose response, and different clinical aspects (Khalili et al., 2020; Zhang et al., 2020).

*Tenofovir* is a real nucleotide analog that has a phosphate group attached to a nitrogenous base (Härter et al., 2020). Once activated, tenofovir acts via different mechanisms, doing a potent reverse transcriptase inhibition and blocking the chain termination in the viral replication. This is an FDA approved drug for HIV and hepatitis C treatment, and it is currently used against the human herpes simplex virus, inhibiting the viral DNA polymerase (McConville et al., 2014). With the ongoing COVID-19 pandemic, this drug has been used in clinical studies with patient concomitant infected with HIV and SARS-CoV-2 (Härter et al., 2020).

*Umifenovir (arbidol)* is an indole-based derivative, which inhibits the influenza virus binding fusion proteins mechanisms (Hemagglutinin) (Blaising et al., 2014). It is a broad-spectrum oral antiviral used for the treatment and prophylaxis of influenza A and B and other respiratory infections (Blaising et al., 2014). As an oral antiviral, it has been used for the treatment and prophylaxis of influenza and other viral respiratory infections, licensed for use in Russia in 1993 and in China since 2006 (Liu et al., 2009; Mani Mishra et al., 2020). This drug has proven effectiveness *in vitro*, *in vivo*, and clinical studies for different viruses, including influenza A and B, Zika, as well as agents of acute respiratory tract infection: adenovirus, respiratory syncytial virus, coronavirus or SARS virus, rhinovirus, parainfluenza virus (Brooks et al., 2012; Tannock et al., 2016; Kadam & Wilson, 2017; Fink et al., 2018; Haviernik et al., 2018; Maleev et al., 2019). Some authors reported that this drug effectively inhibited SARS-CoV-2 when compared to other anti-influenza drugs of therapeutic use (Wang et al., 2020), and it was effective (EC50) in a range of inhibitory concentration against influenza and within a range of maximum plasma concentration estimated to be effective for SARS-CoV-2 (Sun et al., 2013). Currently, umifenovir is under clinical investigation as a potential agent for the treatment and prophylaxis of SARS-CoV-2 infections (Mani Mishra et al., 2020) and the early treatment with this drug can decrease the incidences of pneumonia in a high-risk hospitalized population (Beigel et al., 2019). In addition, it can be suggested that arbidol is associated with a decrease in infection among exposed individuals by COVID-19 (Zhang et al., 2020). *In silico* studies for this drug displayed activity with several SARS-CoV-2 targets, such as Mpro and Spike proteins (Vankadari, 2020; Zhao et al., 2020).

Several of the accepted papers in this review reported drugs with promising results, mainly with *in silico* and *in vitro* studies, which can be considered for further *in vivo* and clinical trials experiments. *Acyclovir* is a nucleoside analog that inhibits the action of viral DNA polymerase and DNA replication of different herpesviruses (O’Brien and Campoli-Richards, 1989); however, it did not show any effect against 2019-nCoV (Li et al., 2020). *Indinavir* is an antiretroviral protease inhibitor used against type 1 HIV infection. *In silico* studies reported that this drug could be used as a probable inhibitor of the SARS-CoV-2 Mpro, according to molecular docking experiments with the crystallized structures 5R7Z, 5R80, 5R81 and 5R82 (Shah et al., 2020). *Pleconaril* is a drug used for prevention of asthma, as well as common cold symptoms in asthmatic individuals exposed to respiratory infections. This drug acts against Picornaviridae viruses, and *in silico* molecular docking experiments indicated that pleconaril could be a SARS-CoV-2 spike protein blocker and may be selected for further preclinical and clinical experiments against this virus (Böcher et al., 2020). *Sofosbuvir* is a nucleoside analog used against HCV infections that acts inhibiting the viral RNA-dependent polymerase, and *in silico* studies demonstrated that this compound could complex with the SARS-CoV2 RNA polymerase (Elfiky, 2020), as well as other viral enzymes (Shah et al., 2020). *Tipranavir* is a non-peptide inhibitor of the HIV protease indicated for combined antiretroviral treatment. This drug has been repurposed in *silico* studies against 3CL SARS-CoV-2 proteases (Böcher et al., 2020). *Zanamivir* is a direct-acting antiviral drug that acts as a neuraminidase inhibitor against influenza A and B (Elfiky, 2020), and *in silico* studies demonstrated interaction with viral transcription proteases against SARS-CoV2 (Shah et al., 2020). Additional studies have been reporting the broad-spectrum anti-parasitic drug *ivermectin* as a SARS-CoV-2 replication inhibitor (Caly et al., 2020), since other studies have reported many antiviral actions against HIV (Wagstaff et al., 2012; Caly et al., 2020; Heidary and Gharebaghi, 2020), DENV (Xu et al., 2018; Caly et al., 2020; Heidary and Gharebaghi, 2020), ZIKA (Caly et al., 2020; Heidary and Gharebaghi, 2020), and Influenza A (Götz et al., 2016; Heidary and Gharebaghi, 2020). This drug is a macrocyclic lactone with its main antiviral mechanism of action in the nuclear transport role of the host importin α (IMPα) protein (Jans and Wagstaff, 2020; Schwemmle et al., 2020). Furthermore, one study has showed that this molecule presented antiviral effects against the SARS-CoV-2 in *vitro* assays with Vero/hSLAM cells with 5000-fold reduction in viral RNA after 48 h with IC50 varying from 2.2 to 2.5 μM (Caly et al., 2020; Sharun et al., 2020). *Teicoplanin* is a glycopeptide antibiotic with its main activity to treat bacterial infection (Reynolds, 1989; Zhou et al., 2016). *In vitro* experiments demonstrated that this drug inhibited cell entry of the EBOV into primary human umbilical vein endothelial cells, A549 cells, HeLa cells, and THP-1 cells with an IC50 of 0.34 μM (Zhou et al., 2016), as well as its derivatives demonstrated antiviral activity against influenza strains, vaccinia, herpes, and human coronavirus (Bereczki et al., 2020). Recently, it was published that teicoplanin potently blocks the HIV-luc/2019-nCoV-S pseudoviruses entry into human A549 cells with a IC50 of 1.66 μM, suggesting this could be caused by the cathepsin L inhibition. Additionally, it was demonstrated that this drug repressed viral entrance into HEK293T and Huh7 cells (Zhang et al., 2020).

A recent study which performed a high-throughput screening using The ReFRAME library for drug repurposing against the SARS-CoV-2, investigated 11,987 FDA approved compounds in infected Vero E6 cell assays, and included a gene set enrichment analysis, which returned that they could be affecting viral replication and dynamics by acting in different targets, such as modulators of benzodiazepine receptors, aldose reductase, potassium channels, cholesterol homeostasis, serine proteases and retinoic acid receptor agonists. Furthermore, the authors realized an orthogonal validation of 300 active compounds with concentrations between 2.5 and 1 μM, and found 100 molecules that were capable to reduce viral replication, as well as several validated by the gene set enrichment analysis target classes. Compound efficacies were additionally checked with Huh-7 and HEK293T cells transduced with angiotensin-converting enzyme 2 (ACE2), and Thirteen compounds exhibited nanomolar EC_50_ values, including a peroxisome proliferator-activated receptor-γ (PPAR-γ) agonist DS-6930, the HIV-1 reverse transcriptase inhibitor R 82913, and the anti-mycobacterial clofazimine. Additionally, it was found that five of most potent inhibitors presented activity in the cell viral entry step but, on the other hand, it is suggestive that the protease inhibitors VBY-825, ONO 5334, Z LVG CHN2 and MDL 28170 are acting in host’s proteases once they have not acted on both SARS-CoV-2 3C-like and papain-like proteases. The compound Z LVG CHN2 is probably acting as an endosomal protease inhibitor whereas ONO 5334 is a cathepsin K inhibitor, and VBY-825 a cathepsin protease inhibitor. Another assay with human induced pluripotent stem cell (iPSC)-derived pneumocyte-like cells indicated that the molecules ONO 5334, MDL 28170 and apilimod drastically reduced the number of infected cells. Furthermore, an *ex vivo* lung culture system assay showed apilimod as a potent antagonist to viral replication in comparison to the positive control remdesivir (Huang et al., 2020; Wec et al., 2020).

A second recent study with a large-scale molecule library, with approximately 1000 FDA approved drugs and 2,100 drug-like molecules with validated pharmacological activity purchased from Selleckchem, performed an antiviral screening using Lung epithelial Calu-3 cells, Vero E6 and Huh7.5 cells. Initially, the authors found that remdesivir and hydroxychloroquine were active in infected Vero E6, with IC_50_ of 0.46 and 1.32 μM, respectively, as well as other six drugs including the natural compound nanchangmycin, with IC_50_ of 0.01 μM. Additionally, Huh7.5 cell assays demonstrated that 33 drugs were active with IC50 below 0.5 μM, including remdesivir, hydroxychloroquine and nanchangmycin. On the other hand, since remdesivir was active in infected Calu-3 cells, hydroxychloroquine and its derivates presented none activity. This suggests that there is a different mechanism of endosomal acidification in this cell types which turns the mechanism of action of the hydroxychloroquine and its derivatives, as well as the drug Z-FA-FMK ineffective in these cells but active in Vero E6 and Huh7.5. Thus, the authors suggested the role of the he plasma membrane-associated serine protease (TMPRSS2), allowing the SARS-CoV-2 entry in Calu-3 cells, and proposed a specific inhibition action of the drug camostat with IC50 of 0.35 μM since it has not presented activity in Vero E6 and Huh7.5 cells (Walker et al., 2020). This mechanism was also demonstrated in another study using human bronchial epithelial cells (HBEC), primary type II alveolar epithelial cells (AECII), and Calu-3 cells for Influenza A and B Virus (Limburg et al., 2019). On the other hand, recent clinical studies with hydroxychloroquine indicated a lack of efficacy in acute infected hospitalized patients with COVID-19, but with small cardiac effects and cardiac deaths (Giovanna and Carlo, 2020; Pan et al., 2020), which could indicate that this drug have its main action as a prophylactic agent, as well as in non-severe cases. Additionally, similar results were achieved for remdesivir, lopinavir (Pan et al., 2020), as well as for the combination between lopinavir and ritonavir (Horby et al., 2020). Thus, this is suggestive that these drugs could be used as prophylactic mechanisms, as well as in the early stages of COVID-19 infection in non-hospitalized patients. Therefore, further prophylactic studies are required for hydroxychloroquine, remdesivir and lopinavir, before they can be largely used by the physicians. Other nine drug candidates presented selective antiviral index greater than 2 against SARS-CoV-2 Calu-3 cells: Salinomycin, Y-320, AZD8055, bemcentinib, dacomitinib, WYE-125132, ebastine, Dp44mT, and cyclosporine (Limburg et al., 2019).

The new coronavirus pandemic caused by the SARS-CoV-2 has provoked a global health and economic crisis in most countries, which has mobilized a great number of scientific research groups in many fields of study, especially with drug therapy and vaccination, in order to find prophylaxis and therapeutic alternatives against the COVID-19. Although many research results have revealed palliative drug treatment against this infection focused in human targets, many other researches have been publishing drug repurposing experiments, using previous experimental data, with main targeting other viruses’ treatments, with *in silico* and *in vitro* experiments. On the other hand, clinical experiments with previously approved drugs against the new coronavirus lack in number of drug options, number of patients in different health conditions and control groups, as well as the time of evaluation, which is understandable, since only less than seven months have passed after the start of the SARS-CoV-2 outbreak. Furthermore, many vaccination options are still in different clinical test phases in many countries, without any guarantee to solve this global problem. Therefore, the use of clinical protocol-based scientific evidence data for SARS-CoV-2 prophylaxis, as well as for the daily routine in hospitals for its treatment, is crucial for controlling the disease spread, prognostic and patient recovery, and can indeed help save many lives.

In this review, we proposed a list of 22 approved drugs and compounds, with relevant clinical data, and *in vivo*, *in vitro* and *in silico* evidences, which can guide prophylaxis studies with large individual groups. Additionally, we included more 22 compounds between FDA approved drugs and drug-like like molecules, which were tested in large-scale screenings, as well as those repurposed approved drugs with new mechanism of actions. Furthermore, this review contributes to avoiding the concomitant use of drugs associated with polypharmacy (many times without scientific evidences), which can lead to serious health side and adverse effects, sometimes with toxic and degenerative drug interactions for humans. The integration between clinical trials data, *in silico*, *in vitro,* and *in vivo* screenings can assist in the rational use of new antiviral drugs not only for the COVID-19 prophylaxis, but also for its treatment, even in more advanced proliferation stages.

## Data Availability Statement

The original contributions presented in the study are included in the article/Supplementary Material, further inquiries can be directed to the corresponding author.

## Author Contributions

BA participated in the manuscript conceptualization, experimental design and writing, and supervision; FR writing and data analysis; NS experimental design, data analysis; AF, WS, and SS writing and data analysis; DB, AG-N, AB, and VA overall manuscript review and English review.

## Funding

This work was supported by Grant from the Coordenação de Aperfeiçoamento de Pessoal de Nível Superior (CAPES) Grant #88887.506611/202-00. Alexander Birbrair is supported by a Grant from Instituto Serrapilheira/Serra-1708-15285, a Grant from Pró-reitoria de Pesquisa/Universidade Federal de Minas Gerais (PRPq/UFMG) (Edital 05/2016), a Grant from CNPQ (Universal, Process No. 405977/2018-2), a Grant from National Institute of Science and Technology in Theranostics and Nanobiotechnology (CNPq/CAPES/FAPEMIG, Process No. 465669/2014-0), a Grant from FAPEMIG (Rede Mineira de Engenharia de Tecidos e Terapia Celular (REMETTEC, RED-00570-16)), a Grant from FAPEMIG (Rede De Pesquisa Em Doenças Infecciosas Humanas E Animais Do Estado De Minas Gerais (RED-00313-16)), and a productivity fellowship from the National Council for Scientific and Technological Development (CNPq).

## Conflict of Interest

The authors declare that the research was conducted in the absence of any commercial or financial relationships that could be construed as a potential conflict of interest.
