# Supplementary material for: Repurposing Approved Drugs for Guiding COVID-19 Prophylaxis: A Systematic Review
Source: Front Pharmacol. 2020 Dec 14;11:590598. doi: 10.3389/fphar.2020.590598 (PMC7772842; doi:10.3389/fphar.2020.590598)
Supplement: Supplementary file 3 [file datasheet1.pdf]

Supplemental material 1

Table 1. Search protocol for systematic literature review with the selection and data recovery criteria.

**Potential antiviral drugs for COVID-19 prophylaxis: A systematic review**<br>**Repurposing approved drugs for guiding COVID-19 prophylaxis: A systematic review**	
Authors	Bruno Silva Andrade[1], Fernanda de Souza Rangel[1,2], Naiane Oliveira Santos[2], Andria dos Santos Freitas[1,2], Wagner Rodrigues de Assis Soares[1,3], Sérgio Siqueira[1], Aristóteles Góes-Neto[3], Vasco Ariston de Carvalho Azevedo[4]
Description	COVID 19 is an ongoing pandemic, caused by the new Severe Respiratory Syndrome, Coronavirus 2 (SARS CoV-2) and presents major challenges in identifying effective drugs for prophylaxis and treatment.
Objective	Identify and analyze the main drugs tested in vitro and in vivo and randomized clinical studies on the use of drugs for pre- and post-exposure prophylaxis in the treatment of COVID-19.
**Research question**	
Question	What are the drugs with prophylactic potential against COVID 19, what is their mechanism of action and toxicity?
Control	Available revisions
Expected results	Primary studies with empirical experiments that test applications of different drugs with prophylactic potential for the treatment of Covid-19.
Primary studies	Published works with drugs with prophylactic potential undergoing in vitro testing, in vivo or case reports.
**Identification of studies**	
Keywords	Covid-19 ou SARS-CoV-2 ou Coronavirus ou 2019 nCoV, prophylaxis, prophylactic, pre-exposure, COVID-19, Sars-Cov-2 Chemoprophylaxis, repurposed, strategies, clinical, trials, anti-sars-cov-2, anti-covid-19, Antiviral, therapy prevention in vitro, in cells e human testing.
Drugs used in the search	Alisporivir, Anisomycin, Benztropine, Dalbavancin, Homoharringtonine, Oritavancin, Telavancin, Clomipramine, Dasatinib, Emetine, Toremifene, Cepharanthine, Promethazine, Imatinib, Indomethacin, Tamoxifen, Amodiaquine, Luteolin, Niclosamide, Emodin, Gemcitabine, Ivermectin, Teicoplanin, Mefloquine, Chlorpromazine, Amiodarone, Glycyrrhizin, Nelfinavir, Camostat, BCX4430 (Galidesivir), Ribavirin, Remdesivir, Arbidol (Umifenovir), Lopinavir, Chloroquine, Hydroxychloroquine, umifenovir e Tenofovir.
**Database / Search Strings**	

PubMed:

0. ((((((Covid-19[Title]) OR SARS-CoV-2[Title]) OR Coronavirus[Title]) OR 2019 nCoV[Title]) AND Prophylaxis[Body - All Words]) AND Alisporivir[Body - All Words]) NOT Review[Abstract]
1. ((((((Covid-19[Title]) OR SARS-CoV-2[Title]) OR Coronavirus[Title]) OR 2019 nCoV[Title]) AND Prophylaxis[Body - All Words]) AND Anisomycin[Body - All Words]) NOT Review[Abstract]
2. ((((((Covid-19[Title]) OR SARS-CoV-2[Title]) OR Coronavirus[Title]) OR 2019 nCoV[Title]) AND Prophylaxis[Body - All Words]) AND Benztropine[Body - All Words]) NOT Review[Abstract]
3. ((((((Covid-19[Title]) OR SARS-CoV-2[Title]) OR Coronavirus[Title]) OR 2019 nCoV[Title]) AND Prophylaxis[Body - All Words]) AND Dalbavancin[Body - All Words]) NOT Review[Abstract]
4. ((((((Covid-19[Title]) OR SARS-CoV-2[Title]) OR Coronavirus[Title]) OR 2019 nCoV[Title]) AND Prophylaxis[Body - All Words]) AND Homoharringtonine[Body - All Words]) NOT Review[Abstract]
5. ((((((Covid-19[Title]) OR SARS-CoV-2[Title]) OR Coronavirus[Title]) OR 2019 nCoV[Title]) AND Prophylaxis[Body - All Words]) AND Oritavancin[Body - All Words]) NOT Review[Abstract]
6. ((((((Covid-19[Title]) OR SARS-CoV-2[Title]) OR Coronavirus[Title]) OR 2019 nCoV[Title]) AND Prophylaxis[Body - All Words]) AND Telavancin[Body - All Words]) NOT Review[Abstract]
7. ((((((Covid-19[Title]) OR SARS-CoV-2[Title]) OR Coronavirus[Title]) OR 2019 nCoV[Title]) AND Prophylaxis[Body - All Words]) AND Clomipramine[Body - All Words]) NOT Review[Abstract]
8. ((((((Covid-19[Title]) OR SARS-CoV-2[Title]) OR Coronavirus[Title]) OR 2019 nCoV[Title]) AND Prophylaxis[Body - All Words]) AND Dasatinib[Body - All Words]) NOT Review[Abstract]
9. ((((((Covid-19[Title]) OR SARS-CoV-2[Title]) OR Coronavirus[Title]) OR 2019 nCoV[Title]) AND Prophylaxis[Body - All Words]) AND Emetine[Body - All Words]) NOT Review[Abstract]
10. ((((((Covid-19[Title]) OR SARS-CoV-2[Title]) OR Coronavirus[Title]) OR 2019 nCoV[Title]) AND Prophylaxis[Body - All Words]) AND Toremifene[Body - All Words]) NOT Review[Abstract]
11. ((((((Covid-19[Title]) OR SARS-CoV-2[Title]) OR Coronavirus[Title]) OR 2019 nCoV[Title]) AND Prophylaxis[Body - All Words]) AND Cepharanthine[Body - All Words]) NOT Review[Abstract]
12. ((((((Covid-19[Title]) OR SARS-CoV-2[Title]) OR Coronavirus[Title]) OR 2019 nCoV[Title]) AND Prophylaxis[Body - All Words]) AND Promethazine[Body - All Words]) NOT Review[Abstract]
13. ((((((Covid-19[Title]) OR SARS-CoV-2[Title]) OR Coronavirus[Title]) OR 2019 nCoV[Title]) AND Prophylaxis[Body - All Words]) AND Imatinib[Body - All Words]) NOT Review[Abstract]
14. ((((((Covid-19[Title]) OR SARS-CoV-2[Title]) OR Coronavirus[Title]) OR 2019 nCoV[Title]) AND Prophylaxis[Body - All Words]) AND Indomethacin[Body - All Words]) NOT Review[Abstract]
15. ((((((Covid-19[Title]) OR SARS-CoV-2[Title]) OR Coronavirus[Title]) OR 2019 nCoV[Title]) AND Prophylaxis[Body - All Words]) AND Tamoxifen[Body - All Words]) NOT Review[Abstract]

16. ((((((Covid-19[Title]) OR SARS-CoV-2[Title]) OR Coronavirus[Title]) OR 2019 nCoV[Title]) AND Prophylaxis[Body - All Words]) AND Amodiaquine[Body - All Words]) NOT Review[Abstract]
17. ((((((Covid-19[Title]) OR SARS-CoV-2[Title]) OR Coronavirus[Title]) OR 2019 nCoV[Title]) AND Prophylaxis[Body - All Words]) AND Luteolin[Body - All Words]) NOT Review[Abstract]
18. ((((((Covid-19[Title]) OR SARS-CoV-2[Title]) OR Coronavirus[Title]) OR 2019 nCoV[Title]) AND Prophylaxis[Body - All Words]) AND Niclosamide[Body - All Words]) NOT Review[Abstract]
19. ((((((Covid-19[Title]) OR SARS-CoV-2[Title]) OR Coronavirus[Title]) OR 2019 nCoV[Title]) AND Prophylaxis[Body - All Words]) AND Emodin[Body - All Words]) NOT Review[Abstract]
20. ((((((Covid-19[Title]) OR SARS-CoV-2[Title]) OR Coronavirus[Title]) OR 2019 nCoV[Title]) AND Prophylaxis[Body - All Words]) AND Gemcitabine[Body - All Words]) NOT Review[Abstract]
21. ((((((Covid-19[Title]) OR SARS-CoV-2[Title]) OR Coronavirus[Title]) OR 2019 nCoV[Title]) AND Prophylaxis[Body - All Words]) AND Ivermectin[Body - All Words]) NOT Review[Abstract]
22. ((((((Covid-19[Title]) OR SARS-CoV-2[Title]) OR Coronavirus[Title]) OR 2019 nCoV[Title]) AND Prophylaxis[Body - All Words]) AND Teicoplanin[Body - All Words]) NOT Review[Abstract]
23. ((((((Covid-19[Title]) OR SARS-CoV-2[Title]) OR Coronavirus[Title]) OR 2019 nCoV[Title]) AND Prophylaxis[Body - All Words]) AND Mefloquine[Body - All Words]) NOT Review[Abstract]
24. ((((((Covid-19[Title]) OR SARS-CoV-2[Title]) OR Coronavirus[Title]) OR 2019 nCoV[Title]) AND Prophylaxis[Body - All Words]) AND Chlorpromazine[Body - All Words]) NOT Review[Abstract]
25. ((((((Covid-19[Title]) OR SARS-CoV-2[Title]) OR Coronavirus[Title]) OR 2019 nCoV[Title]) AND Prophylaxis[Body - All Words]) AND Amiodarone[Body - All Words]) NOT Review[Abstract]
26. ((((((Covid-19[Title]) OR SARS-CoV-2[Title]) OR Coronavirus[Title]) OR 2019 nCoV[Title]) AND Prophylaxis[Body - All Words]) AND Glycyrrhizin[Body - All Words]) NOT Review[Abstract]
27. ((((((Covid-19[Title]) OR SARS-CoV-2[Title]) OR Coronavirus[Title]) OR 2019 nCoV[Title]) AND Prophylaxis[Body - All Words]) AND Nelfinavir[Body - All Words]) NOT Review[Abstract]
28. ((((((Covid-19[Title]) OR SARS-CoV-2[Title]) OR Coronavirus[Title]) OR 2019 nCoV[Title]) AND Prophylaxis[Body - All Words]) AND Camostat[Body - All Words]) NOT Review[Abstract]
29. (((((((Covid-19[Title]) OR SARS-CoV-2[Title]) OR Coronavirus[Title]) OR 2019 nCoV[Title]) AND Prophylaxis[Body - All Words]) AND BCX4430[Body - All Words]) OR Galidesivir[Body - All Words]) NOT Review[Abstract]
30. ((((((Covid-19[Title]) OR SARS-CoV-2[Title]) OR Coronavirus[Title]) OR 2019 nCoV[Title]) AND Prophylaxis[Body - All Words]) AND Ribavirin[Body - All Words]) NOT Review[Abstract]
31. ((((((Covid-19[Title]) OR SARS-CoV-2[Title]) OR Coronavirus[Title]) OR 2019 nCoV[Title]) AND Prophylaxis[Body - All Words]) AND Remdesivir[Body - All Words]) NOT Review[Abstract]

32. (((((((Covid-19[Title]) OR SARS-CoV-2[Title]) OR Coronavirus[Title]) OR 2019 nCoV[Title]) AND Prophylaxis[Body - All Words]) AND Arbidol[Body - All Words])OR Umifenovir[Body - All Words])NOT Review[Abstract]
33. ((((((Covid-19[Title]) OR SARS-CoV-2[Title]) OR Coronavirus[Title]) OR 2019 nCoV[Title]) AND Prophylaxis[Body - All Words]) AND Lopinavir[Body - All Words]) NOT Review[Abstract]
34. ((((((Covid-19[Title]) OR SARS-CoV-2[Title]) OR Coronavirus[Title]) OR 2019 nCoV[Title]) AND Prophylaxis[Body - All Words]) AND Chloroquine[Body - All Words]) NOT Review[Abstract]
35. ((((((Covid-19[Title]) OR SARS-CoV-2[Title]) OR Coronavirus[Title]) OR 2019 nCoV[Title]) AND Prophylaxis[Body - All Words]) AND Hydroxychloroquine[Body - All Words]) NOT Review[Abstract]
36. ((((((Covid-19[Title]) OR SARS-CoV-2[Title]) OR Coronavirus[Title]) OR 2019 nCoV[Title]) AND Prophylaxis[Body - All Words]) AND umifenovir[Body - All Words]) NOT Review[Abstract]
37. ((((((Covid-19[Title]) OR SARS-CoV-2[Title]) OR Coronavirus[Title]) OR 2019 nCoV[Title]) AND Prophylaxis[Body - All Words]) AND Tenofovir[Body - All Words]) NOT Review[Abstract]

Scopus:

38. ABS (Prophylaxis OR prophylactic AND pre-exposure” AND COVID-19 OR Sars-Cov-2)
39. ABS (Chemoprophylaxis AND pre-exposure AND COVID-19 OR Sars-Cov-2)
40. ABS (repurposed AND prophylaxis AND covid-19 OR sars-cov-2)
41. ABS (chemoprophylaxis OR prophylaxis AND strategies AND clinical AND trials AND covid-19 OR sars-cov-2)
42. ABS (anti-sars-cov-2 OR anti-covid-19 AND in vitro)
43. ABS (prophylaxis AND antiviral AND therapy AND covid-19 OR sars-cov-2)
44. ABS (prophylaxis OR prevention AND covid-19 OR sars-cov-2 AND "in vitro" OR "in cells" OR "human testing")
45. ABS (repurposed AND chemoprophylaxis AND covid-19 OR sars-cov-2)

**Criterion**	
Languages	English
Sources	PudMed and Scopus
Kind of study	Data Articles and Research Articles
Initial selection	Based on the title, abstract and keywords.
Final selection	Based on the inclusion and exclusion criteria.
Inclusion and exclusion criteria	Inclusion:<br>Articles in English language and discuss drugs with prophylactic potential for COVID 19.<br><br>Exclusion:<br>- Review articles, which are not aligned with the object of study, case study, clinical guidelines, research strategies, short communications and unfinished studies.
Data extraction strategy	Drugs used, testing phases (I, II and III), drug efficacy, half-life, number of patients or cells used in the tests, drug toxicity, interactions between drugs and side effects.

Data summarization	The collected data will be demonstrated in tables, figures and graphs.
