# Supplementary figures and images for: Repurposing Approved Drugs for Guiding COVID-19 Prophylaxis: A Systematic Review
Source: Front Pharmacol. 2020 Dec 14;11:590598. doi: 10.3389/fphar.2020.590598 (PMC7772842; doi:10.3389/fphar.2020.590598)

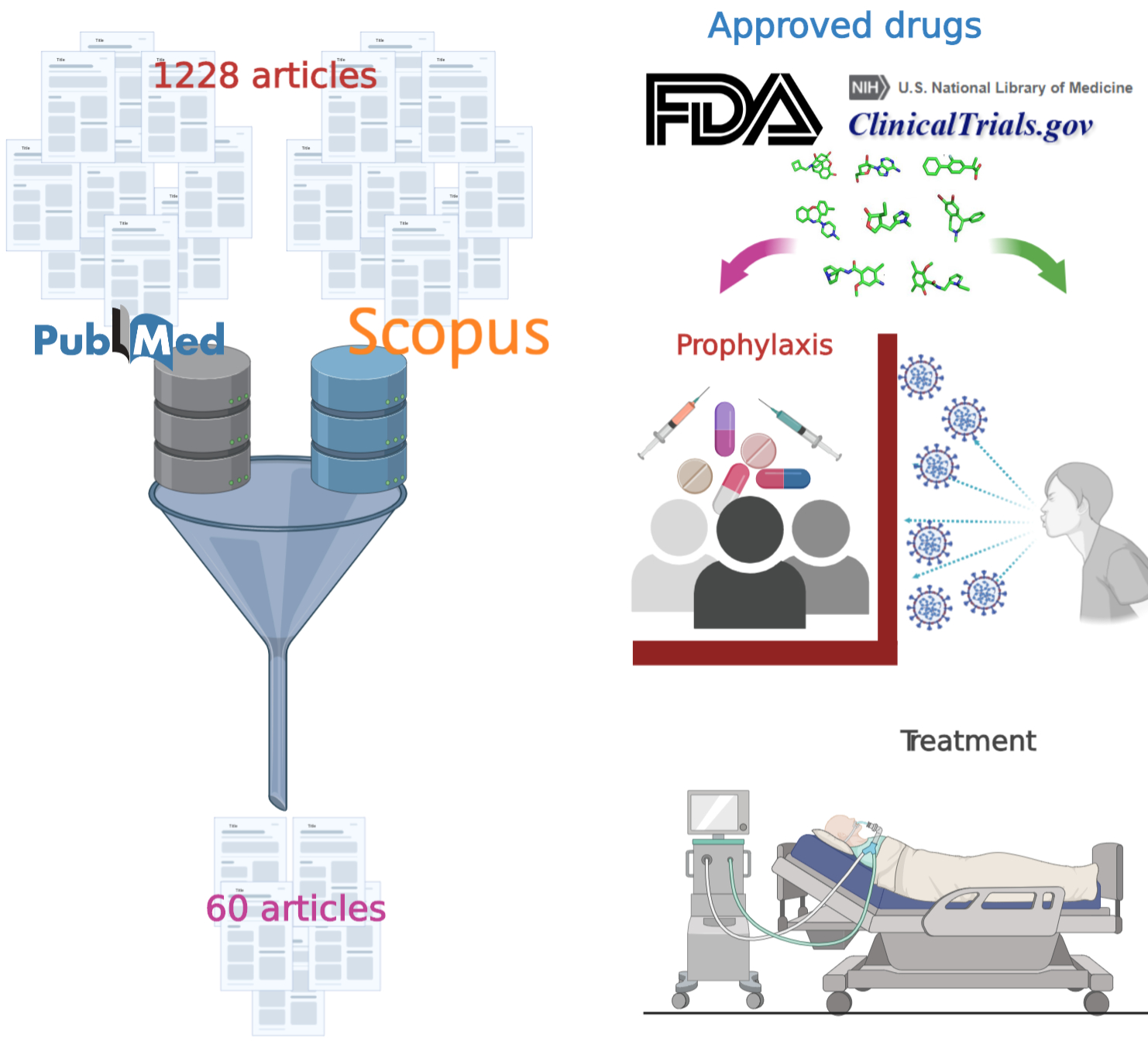

Supplement: Supplementary file 4 [file image1.jpeg]
